# Pediatric unsedated transnasal endoscopy: applications, equipment, and future directions

**DOI:** 10.3389/fped.2025.1585705

**Published:** 2025-05-09

**Authors:** Paroma Bose, Ryan Pitman

**Affiliations:** Division of Pediatric Gastroenterology, Hepatology, and Nutrition, Department of Pediatrics, Indiana University School of Medicine, Indianapolis, IN, United States

**Keywords:** unsedated endoscopy, transnasal endoscopy, GERD (gastroesophageal reflux disease), EoE (eosinophilic esophagitis), EGD (esophagogastroduodenoscopy)

## Abstract

Gastrointestinal (GI) endoscopy is a valuable tool to diagnose and treat GI conditions. Traditional pediatric GI endoscopy uses sedation or general anesthesia, with associated risks of cardiopulmonary compromise and social and economic costs like school or work absence. Unsedated, transnasal endoscopy is an approach that mitigates these disadvantages but provides similar diagnostic benefit to conventional endoscopy. Ongoing advances in the field of pediatric transnasal endoscopy will be driven by an enhanced understanding of current indications, available equipment, procedural comfort strategies, and recent developments in new diagnostic and therapeutic uses.

## Introduction

In pediatrics, gastrointestinal (GI) endoscopy is a valuable tool to assess GI symptoms, diagnose mucosal diseases, and provide therapeutic interventions. Pediatric endoscopy is often done with sedation or general anesthesia for patient comfort and procedural safety (1). Cardiopulmonary compromise is the most common adverse event (AE) in pediatric endoscopy with a higher risk with intravenous sedations compared to general anesthesia (2). Additionally, anesthesia may lead to activity limitations and delayed return to work or school. Unsedated (“u”) GI endoscopy, more specifically upper endoscopy or esophagogastroduodenoscopy (EGD), is an alternative method to sedated (“s”) endoscopy that is safe, well-tolerated, and with expanding clinical uses. Though conventional pediatric EGD uses a peroral (“P”) approach, transnasal (“TN”) endoscopy is gaining popularity. In this article, we aim to review the history of TN endoscopy, scopes available for use and their features, anesthetic regimens, current applications in children and adults, and future directions.

## History of unsedated and transnasal GI endoscopy

The earliest report of successful unsedated GI endoscopy documented completion of uP-EGD in 187 of 200 adult patients seen in a one-visit clinic for dyspepsia in the 1970s (3). Esophagitis, gastritis, duodenitis, hiatal hernia, and other abnormalities were identified with this approach. 60% of patients found the procedure mildly unpleasant, and 5.5% of procedures were aborted due to inability to tolerate the procedure (3). A larger prospective study from the 1980s reported 2,000 consecutive successful uP-EGD in patients (8–86 years) with only 1.6% of patients requiring IV sedation in order to complete the procedure (4). Conventional P-EGD typically uses endoscopes of 9.2–9.9 mm diameter.

The volume of publications about unsedated endoscopy increased in the 1990s with emergence of the TN approach. Esophageal intubation with a bronchoscope (5.3 mm diameter) transnasally was first introduced in 1991 in adult patients undergoing evaluation for cervical dysphagia (5). The investigator then performed successful uTN-EGD on 20 healthy adult volunteers with topical nasal xylocaine gel and pharyngeal cetacaine spray (5). A 1995 study (6) compared uTN-EGD to P-EGD with or without general anesthesia within the same patient in 24 adults. Eighteen required IV conscious sedation for sP-EGD, while 6 did not. These patients reported significantly higher acceptability of uTN-EGD compared to uP-EGD and sP-EGD, with fewer symptoms of choking sensation, sore throat, and discomfort. There was overall agreement between endoscopic findings between the uTN-EGD and P-EGD (6). A 1998 study (7) showed similar efficacy and tolerance of uTN-EGD vs. sP-EGD and estimated that 12 TN-EGDs could be performed in the same time as 9 P-EGDs with 65% decrease in consumable and medication costs and 92% decrease in time in procedure and recovery area (7).

Though a majority of historical studies of unsedated endoscopies are adult studies, a 2002 study (8) specifically explored feasibility of unsedated endoscopy in children. Twenty-seven sP-EGDs (mean 12.2 years ± 2.7) were compared to 21 uP-EGDs (mean 13.5 years ± 2.7) using a 9.8 mm diameter endoscope with similar success rate between the two groups at 96.3% and 95.2%, respectively. There was no significant change in anxiety scores before and after uP-EGD. Anxiety scores were higher in the sP-EGD group but reduced post procedure, and so authors suspected that anxious children were more likely to request sedation. Children undergoing sP-EGD spent twice as long in the endoscopy suite compared to uP-EGD (8).

With growing interest in the TN approach came the development of ultra-thin scopes for GI use. Early studies in healthy adults using endoscopes with 5.9–6.0 mm diameter demonstrated equivalence of the quality of GI assessment between uTN-EGD and uP-EGD (9). Both routes of insertion were generally well-tolerated, but with variable results on pain/discomfort and willingness to repeat the procedure (9–11). Using an ultra-slim scope for uP-EGD significantly reduced direct and indirect costs compared to sP-EGD (12).

Decreasing scope size to improve comfort with TN approach has also been explored, and procedure success is higher with a smaller scope. For example, a large study of 1,100 adults compared uTN-EGD using scopes with diameters of 5.9–5.3 mm (13). Overall, procedure success was 93.9%. The larger scope diameter was a predictor of procedure failure and was also more associated with epistaxis or nasal pain. 95.2% (982/1,100) said they would elect to do uTN-EGD again (13).

## Transnasal endoscopy equipment

Recent advances in ultra-thin endoscopes have driven the interest in TN endoscopy in children. Early reports of uTN-EGD in adults using “ultra-thin” adult and pediatric endoscopes (5.3–6.0 mm diameter) (5, 6) had limited application to children due to their smaller nasopharyngeal anatomy (14). Thus, the first reports of pediatric uTN esophagoscopy (uTN-E) utilized even smaller diameter reusable pediatric bronchoscopes (4.1 mm) (14–16). Flexible pediatric bronchoscopes are an attractive option for uTN-E with a small external diameter (3.7–4.2 mm) and a single channel (1.2–2.0 mm) for insufflation of air and biopsy forceps for tissue sampling. Adequacy of biopsies samples has been established despite the small working channel of current pediatric bronchoscopes (14, 15, 17). Further, care of reusable pediatric bronchoscopes match established facility protocols for endoscopy processing and maintenance. The utility of pediatric bronchoscopes for TN-EGD is limited by their short length (60 cm), 2-way tip deflection (instead of 4-way deflection), and a single channel for air/water/suction and biopsy instruments. Reusable pediatric bronchoscopes are also limited due to their fragile nature and potential need for repair. Recently, single-use ultra-thin bronchoscopes were introduced to streamline endoscopy efficiency and minimize risk of infectious complications of multi-use endoscopy equipment. While there are no current reports of the use of single-use bronchoscopes for pediatric uTN-E, these may be good options for uTN-E at some institutions.

Gastroscopes are superior to bronchoscopes for uTN-E/EGD due to longer length, 4-way tip deflection, and buttons for insufflating the upper GI lumen, suctioning secretions, and instilling water. The larger working channel (2.2 mm) ensures adequate biopsies specimens and may allow use of interventional equipment. However, the smallest neonatal pediatric gastroscope has an external diameter of 5.8 mm, limiting its use in pediatric uTN-EGD. Dedicated single-use endoscopes have been developed to address the limitations of gastroscopes and bronchoscopes (18). The EvoEndo endoscopy system has multiple length options to perform uTN-E or uTN-EGD (85 or 110 cm) with a small external diameter (3.5 mm) and adequate internal working channel (2 mm) (18). A wide variety of endoscopes are available for use in TN endoscopy (Table 1). Further advancements in reusable and single-use endoscopes will enhance patient tolerance, endoscopic visibility, and facilitate interventional techniques in pediatric TN endoscopy.

**Table 1:** Endoscopes available for transnasal endoscopy.

Manufacturer	Model	Length (cm)	Outer diameter (mm)	Air suction water	Working channel (mm)	Field of vision
Single-use bronchoscopes						
Ambu	aScope 4 Slim	60	4.3	Air	1.2	85°
Ambu	aScope 4 Regular	60	5.4	Air	2	85°
Ambu	aScope 5 HD 5.0/2.2	60	5.7	Air	2.05	120°
Ambu	aScope 5 HD 4.2/2.2	60	4.8	Air	2.15	120°
Ambu	aScope 5 HD 2.7/1.2	60	3.2	Air	1.15	120°
Pentax	ONE Pulmo	60	5.5	Air	2.9	120°
Verathon	bFlex 2 Ultraslim 2.8	56.6	2.8	NA	NA	85° (H/V), 120° (D)
Verathon	bFlex 2 Ultraslim 2.8	56.6	3.8	Air	1.2	85° (H/V), 120° (D)
Verathon	bFlex 2 Ultraslim 2.8	56.6	5	Air	2.2	85° (H/V), 120° (D)
Verathon	bFlex 2 Ultraslim 2.8	56.6	5.8	Air	3	85° (H/V), 120° (D)
Boston Scientific	Exalt Model B Slim	60	4.3	Air	1	90°
Boston Scientific	Exalt Model B Regular	60	5.5	Air	2	90°
Olympus	H-SteriScope Zero	60	2.3	NA	NA	110°
Olympus	H-SteriScope Slim	60	3.3	Air	1.2	110°
Olympus	H-SteriScope Normal	60	4.8	Air	2.2	110°
Olympus	H-SteriScope Large	60	5.7	Air	2.8	110°
Single-use gastrointestinal endoscopes						
EvoEndo	EvoEndo Model LE 85	85	3.5	A/W/S	2	120°
EvoEndo	EvoEndo Model LE 120	120	3.5	A/W/S	2	120°
Reusable bronchoscopes						
Olympus	BF-XP-190	60	3.1	Air	1.2	110°
Olympus	BF-MP190F	60	3.7	Air	1.7	90°
Olympus	BF-P190	60	4.2	Air	2	110°
Olympus	BF-Q190	60	4.8	Air	2	120°
Olympus	BF-H190	60	5.5	Air	2	120°
Pentax	EB11-J10	60	4.9	Air	1.2	120°
Fujifilm	EB-710P	60	4.1	Air	2	120°
Fujifilm	EB-530P	60	5.1	Air	1.2	120°
Fujifilm	EB580S	60	5.3	Air	2.2	120°
Fujifilm	EB580T	60	5.9	Air	2.8	120°
Fujifilm	EB530H	60	5.4	Air	2	120°
Reusable gastrointestinal endoscopes						
Olympus	BF-XP-190	110	5.8	A/W/S	2.2	140°
Fujinon	EG-740N	110	5.8	A/W/S	2.4	140°

## Topical anesthesia

While general anesthesia is not used during uTN endoscopy, topical anesthesia of the nasopharynx and oropharynx is commonly used to decrease the gag reflex and minimize discomfort (14). A wide range of topical anesthetics have been described with most regimens including lidocaine applied to the nasopharynx/posterior oropharynx by spray or transcatheter application, as well as lidocaine jelly applied to the anterior nares and nasopharynx (19). Unfortunately, there are no prospective comparison studies of topical anesthetic regimens during uTN endoscopy. Vasoconstricting medications including epinephrine, oxymetazoline, naphazoline, phenylephrine as nasal decongestants are variably reported in the uTN endoscopy literature, and some authors suggest that nasal decongestants enhance uTN endoscopy performance and tolerability (20, 21). Prospective evaluations of anesthetic regimens in pediatric flexible laryngoscopy have not demonstrated significant improvement in patient tolerance with topical nasal anesthetics, while nasal decongestant improves both tolerance and visibility of the nasal turbinates (22, 23). Table 2 summarizes the anesthetic regimens reported in both adult and pediatric uTN endoscopy. Topical anesthesia is distasteful, stimulating, and may contribute to discomfort during TN endoscopy (24). Some centers add flavoring to the lidocaine preparation or allow patients to lick a lollipop during anesthesia administration. Anxiolytic medications are not commonly used and may cause disinhibition, compromising the ability to complete the procedure safely. Many efforts have been made to improve patient experience and cooperation and decrease anxiety. A child life specialist can enhance the patient and family experience with calming techniques or visual distraction including access to visual media. Virtual reality systems have been utilized as an effective distraction tool in pediatric uTN endoscopy, however, not all patients elect to utilize virtual reality goggles (25). Prospective studies investigating anesthetic protocols, including medication regimens and routes of administration, are needed to advance the broader adoption of uTN endoscopy technology.

**Table 2:** Topical nasal anesthetic regimens for unsedated endoscopy.

Anesthetic spray	Anesthetic gel	Decongestant	Notes	References
Adult patients				
No intranasal anesthesia				(64)
5% L				(65)
	2% L		Gel on outside of catheter and applied for 5 min	(54, 66–69)
4% L	2% L			(20, 21, 70)
2% L		0.5% Phenylephrine		(71)
4% L		1% Phenylephrine		(11)
4% L		0.5% Oxymetazoline		(72)
4% L		0.05 Oxymetazoline		(73)
5% L		0.5% Phenylephrine		(74)
10% L		0:1% Epinephrine/1% cocaine solution (1:1)		(75)
	2% L	0.05% Naphazoline		(76)
	4% L	0.05 Oxymetazoline		(19)
4 ml of 20 mg/ml Viscous lidocaine		0.5 mg/ml Naphazoline nitrate		(10)
Tetracaine HCL and polidocanol		0.15 mg Oxymetazoline		(77)
0.40% L	2% L	0.5% Naphazoline		(78, 79)
4% L	2% L	0.1% Xylometazoline		(21)
8% L	2% L	0.15 ml Naphazoline		(80)
8%–10% L	2% L	0.05% Epinephrine		(5)
8%–10% L	2% L	0.05 Oxymetazoline		(81)
10% L	2% L	0.05% Oxymetazoline		(82)
10% L	2% L	0.05% Epinephrine		(83)
Pontocaine		Neosynephrine	20% Benzocaine oral spray	(84)
Pediatric patients				
4% L				(14)
	2% L			(18)
4% L			20% Benzocaine oral spray	(16)

L, lidocaine.

## Clinical uses of transnasal GI endoscopy

There are many clinical uses of uTN GI endoscopy in adults including pre-operative assessment for bariatric surgery candidates and screening and monitoring of Barrett's esophagus or esophageal varices. In pediatrics, uTN-E is primarily used to assess esophageal conditions such as eosinophilic esophagitis and surveillance of esophageal atresia. uTN-E is well tolerated in children, and tolerance is not impacted by age, gender, diagnosis of anxiety, ADHD, or autism (26). We discuss advances in TN endoscopy in adults and children below, with particular relevance to pediatric gastroenterology.

### Eosinophilic esophagitis

Eosinophilic esophagitis (EoE) is a chronic esophageal disorder that requires endoscopy for diagnosis and monitoring, thus an ideal condition for TN endoscopy (27). A 2016 pediatric study (14) showed successful uTN-E using a 4.0 mm bronchoscope on 21 patients with EoE ages 8–17 years with no AE. 90.5% of parents and 81% of children were highly satisfied with the procedure, and 100% and 76.2% would elect to do uTN-E again, respectively. Endoscopic findings had high correlation with histologic findings in 85.7% of cases. Total charges for each patient undergoing uTN-E were 60.1% ± 10.7% less than those of their previous sP-EGD (14).

A larger 2019 pediatric study (25) reviewed 294 uTN-Es (including 1 uTN-EGD) in 190 patients ages 3–22 years, with many subjects undergoing multiple uTN-Es to assess EoE treatment changes. While the most common AEs were epistaxis (3.7%) and vomiting (2.7%), a majority (89.8%) experienced no AE. Full thickness epithelium was present in 88% of biopsies obtained with 1.2 mm biopsy forceps compared to 94% in 2.0 mm biopsy forceps. There was an estimated 53.4% reduction in total charges for uTN-E compared to sP-EGD at the time of the study (25).

Since TN-E does not require sedation, the procedure can be repeated in the same patient in succession quickly. In a small study of 6 children ages 11–18 years with EoE treated with diet elimination, uTN-E was repeated 2–4 weeks after food reintroductions. Four patients had active EoE at these early intervals and also had active EoE at a traditional 6-week TN-E (28). TN-E is feasible, effective, and saves cost for children and may allow for more precise management of diet elimination in EoE.

### Esophageal atresia

Esophageal atresia (EA) is a congenital malformation of the esophagus requiring surgical treatment. Children born with EA are at increased risk of gastroesophageal reflux disease, esophagitis, and esophageal strictures and are recommended to have surveillance endoscopies periodically through childhood and into adulthood (29). A case series described four patients with EA who underwent successful uTN-E for surveillance or monitoring of reflux esophagitis and EoE (30). uTN-E demonstrated a variety of findings including normal screening endoscopy, identification of new esophagitis, and visual and histologic remission of esophagitis and EoE after treatment (30).

### Barrett’s esophagus

Barrett's esophagus (BE), which is a transformation of esophageal squamous epithelium to intestinal columnar epithelium, is a risk factor for esophageal adenocarcinoma. BE is associated with gastroesophageal reflux disease (GERD), occurring in 5%–15% of patients with longstanding symptoms (31). Esophageal endoscopic evaluation is the gold standard to assess for BE, traditionally with P-EGD (32, 33). uTN-E or uTN-EGD may serve as a well-tolerated and effective screening for Barrett's esophagus and dysplasia. A 2006 study (34) evaluating detection of Barrett's esophagus and dysplasia in adult patients with chronic GERD demonstrated no difference in BE between uTN-EGD or sP-EGD among 116 patients who completed both procedures. Nine cases of BE were identified with uTN-EGD but missed on sP-EGD, while 5 cases of BE were identified with sP-EGD and missed on uTN-EGD. Biopsy specimens from sP-EGD were significantly larger than those obtained via uTN-EGD (2.60 vs. 1.39 mm^2^, *p* < 0.001). Though anxiety, pain, gagging and choking were more frequent in the uTN-EGD group, discomfort overall was reported as “mild”. Seventy-one percent preferred to have uTN-EGD again over sP-EGD (34).

In a 2023 systematic review and metanalysis of 623 patients who each underwent both uTN-EGD and sP-EGD, pooled sensitivity and specificity were high for detecting intestinal epithelium and intestinal metaplasia with uTN-EGD (35). Patient tolerance was higher with uTN-EGD compared to P-EGD in 3 studies, but no different in 3 other studies. Procedure completion rate was the same between both procedure types, and overall AE rate was low (2.0%). The most common AEs with uTN-EGD were epistaxis and vasovagal symptoms (35).

BE is rare in children compared to adults with prevalence of 0.25%–4.8% (36, 37). The mean duration of reflux symptoms prior to BE diagnosis is 5.3 years, and additional risk factors include neurodevelopmental disorders, congenital esophageal atresia, hiatal hernia, high BMI, and older age (36–39). Due to its low prevalence, there are no consensus guidelines on screening for BE in children with GERD, though endoscopy and biopsy remain the primary tool for detection. These adult studies suggest uTN-EGD may be an effective tool to detect complications of GERD, which could be extrapolated to pediatrics.

### Varices

Adult patients with liver cirrhosis undergo screening endoscopy to evaluate for esophageal varices, and those with large varices are prescribed prophylactic treatment given the high risk of morbidity and mortality from variceal bleeding (40). Sedation in this population poses risk of encephalopathy (41), thus making unsedated endoscopy appealing. A 2002 study (42) of uTN-EGD followed immediately by sP-EGD in 15 adult patients with cirrhosis and no history of variceal bleeding found esophageal varices in 66.7% in both procedures. Identification of gastric varices, large esophageal varices, small esophageal varices, and esophageal rings was concordant between the two procedures. Portal hypertensive gastropathy was identified in one additional patient by sP-EGD compared to uTN-EGD, and Barrett's esophagus was identified in one additional patient by uTN-EGD compared to sP-EGD. No interventions were performed, and procedural tolerance was similar between procedure types. A larger study of 40 adult patients with compensated cirrhosis without active varices prophylaxis underwent successful endoscopic surveillance with uP-EGD (43). Grade 1 varices (17.5%), grade 2 varices (20%), and portal hypertensive gastropathy (22.5%) were identified. Two patients underwent subsequent variceal band ligation with identical findings on sP-EGD to uP-EGD (43). Literature on transnasal pediatric varices assessment is limited. In a 2023 report, a 13-year-old with end-stage renal disease and an 18-year-old with autoimmune hemolytic anemia and autoimmune hepatitis with hepatosplenomegaly required screening for esophageal varices but were deemed high-risk for anesthesia. These patients successfully underwent uTN-EG and uTN-E (respectively) that did not show esophageal varices (44). Unsedated endoscopy may be a safe way to evaluate for varices in populations at high risk of complications from sedation. uTN endoscopy may be better tolerated than uP-EGD and is just as effective at detecting esophageal varices, though endoscopic interventions are limited.

### Obesity

Endoscopic evaluation of the upper GI tract is recommended prior to bariatric surgery in adults to evaluate for conditions that may inhibit surgery success (45). Patients with overweight and obese status, and comorbid complications like sleep apnea, may have higher risk of cardiopulmonary events related to sedation (46), thus unsedated endoscopy is a reasonable approach to pre-operative evaluation. In a small study of 25 adult patients undergoing pre-operative endoscopy for bariatric surgery, all patients tolerated uTN-EGD, and 56% had abnormalities identified including hiatal hernia, gastritis, esophagitis, BE, gastric polyps, gastric ulcer, and esophageal varices (47). In a 2020 study (48) of 94 adult patients who underwent uTN-EGD at a bariatric center, the procedure was successful in 98.9% of patients with endoscopic abnormalities in 88.3% and actionable abnormalities in 23.4%, including hiatal hernia, peptic ulcers, esophagitis, gastritis, varices, and subepithelial lesions. Biopsies obtained in 80.8% were adequate for pathology evaluation. A majority of patients tolerated the procedure well with 84% reporting minimal discomfort, and all patients agreed to repeat uTN-EGD if needed (48).

Preoperative endoscopic evaluation may be of value in children as well. In a 2021 study, 80 children ages 12–18.1 years underwent sP-EGD before bariatric surgery, and 54% had abnormalities identified as esophagitis, gastritis including helicobacter pylori, and duodenitis. Seventy-seven percent were prescribed medical therapy for these findings prior to bariatric surgery (49). A 2024 study reviewed 244 patients aged 9–25 years who had sP-EGD before bariatric surgery; the findings of which affected medical or surgical management in 24.6% (60 cases) (50). Adults and children with overweight or obese status may benefit from GI evaluation with uTN-EGD to optimize preoperative care while reducing risk of complications from sedation.

## Advanced unsedated endoscopy and future directions

In addition to diagnostic uTN-E and uTN-EGD as described above (18), the ultra-thin endoscopes can be used for a broad range of other diagnostic and therapeutic approaches to unsedated endoscopy. Trans-gastrostomy EGD to evaluate the upper GI mucosa in children with gastrostomy can be performed with an ultra-thin endoscope (51) with adequate visualization and biopsy sampling. Ultra-thin endoscopes can also be used to identify aspiration by pediatric flexible endoscopic evaluation of swallowing (FEES) in children with dysphagia (52). Further, uTN-E can be used for esophageal varices screening in children with cirrhosis and portal hypertension (44). While sedated EGD has a limited role in the assessment of gastrointestinal motility, uTN-esophagogastroscopy (EG) may be able to help identify patients with delayed gastric emptying by repeating uTN-EG at defined time intervals after ingestion of a standard meal (53) or by assessing esophageal motor function with swallowing during uTN-E (54). Placement of esophageal Bravo pH probe can also be guided by uTN-E for monitoring of esophageal acid exposure (55).

Additional uTN-EGD interventional procedures have been described in the adult literature including percutaneous gastrostomy placement (56), nasoenteral feeding tube placement (57), lower esophageal sphincter botox injection (58), balloon dilation of esophageal strictures (59), and polypectomy of gastric polyposis (60). uTN-EGD has been proposed as a screening method to identify patients with upper GI bleeds amenable to treatment during sP-EGD (61, 62). As the number of skilled providers performing pediatric uTN-EGD increases, the opportunity for interventional unsedated endoscopy will increase, particularly in children who are at increased risk of complications related to anesthesia.

The availability of ultra-thin TN endoscopy equipment may expand the use of TN endoscopy to initial in-office evaluations of the upper GI tract in children with abdominal complaints with low likelihood of needing endoscopic intervention. A comparison study of uTN-EGD to sP-EGD in 15 adolescents with undifferentiated abdominal pain demonstrated decreased procedure time, anesthesia use, and costs with similar diagnostic outcomes (63). Additional studies are required to determine the utility of in-office uTN-E as a primary method for evaluating a gastrointestinal complaint.

## Conclusion

uTN endoscopy is a cutting-edge technique with capabilities of diagnosing and monitoring upper GI conditions without sedation or anesthesia. With the development of specific TN GI equipment, more pediatric GI physicians learning this technique, and more patients and families experiencing the procedure, it is likely that TN endoscopy will continue to gain popularity in pediatrics.
